# Integrated Physiological, Transcriptomic, and Metabolomic Analyses Reveal the Adaptive Response of *Buchloe dactyloides* to Polycyclic Aromatic Hydrocarbon Stress

**DOI:** 10.3390/plants15152315

**Published:** 2026-07-28

**Authors:** Yuancheng Wang, Donglei Wu, Ao Li, Haolong Xu

**Affiliations:** 1 Zhejiang Key Laboratory of Ecological Environmental Damage Control and Value Transformation, Ecological and Environmental Science and Research Institute of Zhejiang Province, Hangzhou 310007, China; wangyuancheng@zju.edu.cn; 2 College of Environmental and Resource Science, Zhejiang University, Hangzhou 310058, China; 3 Research Institute of Forestry, Chinese Academy of Forestry, Beijing 100091, China

**Keywords:** biosynthesis of flavonoid, metabolism of carbohydrates, metabolic pathways, PAHs

## Abstract

Understanding how plants respond to polycyclic aromatic hydrocarbons (PAHs) stress is essential for evaluating ecological risks and improving phytoremediation strategies. PAHs are widespread and persistent environmental pollutants that exert toxic effects on plants at different developmental stages. Although *Buchloe dactyloides* (Nutt) Engelm shows potential for phytoremediation of PAHs contamination, its root defense mechanism against PAHs remains unclear. To this end, transcriptomics and non-targeted metabolomics were used to study the changes in gene expression and metabolite profiles in roots under PAHs stress. After 70 days of PAHs exposure, *B. dactyloides* roots exhibited increased activities of catalase (CAT) (from 1.955 to 6.436; ca. 3.29) and peroxidase (POD) (from 94.507 to 124.901; ca. 1.32), higher levels of ascorbate (AsA) (from 7976.69 to 18,950.09; ca. 2.38) and glutathione (GSH) (from 21.08 to 37.23; ca. 1.77), and accumulation of proline (from 40.585 to 66.671; ca. 1.64). Significant differences in genes and metabolites were observed between the treatment and control groups, with a total of 4083 differentially expressed genes (DEGs) and 100 differentially accumulated metabolites (DAMs). Further comprehensive analysis of transcriptomics and metabolomics revealed the potential role of multiple pathways in the defense response of *B. dactyloides* roots against PAHs stress, including amino acid synthesis, flavonoid biosynthesis, galactose metabolism, glycerophospholipid metabolism, and other pathways. These pathways may contribute to antioxidative defense under PAHs stress. In addition, increased trehalose and soluble sugar contents likely supplied energy and osmoprotective functions under stress. These findings provide insights into the mechanisms of root adaptation to PAHs and may support the long-term phytoremediation potential of *B. dactyloides*.

## 1. Introduction

Polycyclic aromatic hydrocarbons (PAHs), as persistent organic pollutants, display high soil adsorption coefficients and demonstrate limited biodegradability in environmental matrices; common PAHs include phenanthrene, pyrene, and benzo[a]pyrene. PAHs exhibit high toxicity to a wide range of living organisms and are particularly notorious for their carcinogenic and mutagenic effects [1]. PAHs are pollutants that stimulate stress responses in plant tissues [2]. Shen et al. found that with the accumulation of phenanthrene in wheat leaves, the chlorophyll content of wheat leaves decreased, subcellular structures, and organelles were damaged [3]. Previous research has demonstrated that elevated PAHs concentrations can inhibit the growth of Bermuda grass (*Cynodon dactylon* (L.) Pers.) [4]. Although phytoremediation has proven effective for managing PAHs contaminated soils, its success is contingent upon the plant’s inherent stress resistance mechanisms. The escalating issues of pollution and environmental interference have heightened the need to elucidate plant abiotic stress responses to PAHs, which is vital for agricultural, silvicultural, and ecosystem management.

Environmental stress is a primary trigger for the plant stress response. The PAHs can be absorbed and accumulated by plants, causing disruption of metabolic homeostasis [5]. The accumulation of phenanthrene, pyrene, and benzo[a]pyrene in rice leaves disrupted chlorophyll metabolism, which reduced PSII efficiency and chlorophyll content, thereby inhibiting carbon fixation and biomass accumulation, and ultimately affecting farmland ecosystems [6]. Roots are the primary organs for plants to absorb water and mineral nutrients from the soil. Roots are the primary interface between the plant and soil contaminants and often represent the first tissues exposed to soil-borne toxicants. The absorption of phenanthrene and pyrene by *Lolium multiflorum* Lamk roots involves initial adsorption to the cell wall followed by diffusion to organelles, where transpiration and cellular lipid content are the main drivers of subcellular distribution [7]. Wheat roots actively absorb phenanthrene from contaminated soil and water [8]. Previous studies have found that the PAHs content in the underground parts of corn (*Zea mays* L.) and sunflowers (*Helianthus annuus* L.) is generally higher than that in the aboveground parts [9]. Consequently, the adverse effects of PAHs stress are likely to be more pronounced in roots than in other plant organs. Phenanthrene accumulation induces a large amount of reactive oxygen species (ROS) in roots, enhances lipoxygenase activity and malondialdehyde concentration, leads to lipid peroxidation, and regulates the expression of *CDK* and *CDC2* genes to affect the occurrence and growth of lateral roots [10]. Root response to stress is a primary determinant of overall plant growth and development.

Transcriptomic and metabolomic approaches offer powerful tools for deciphering the intricate processes of root adaptation to PAHs stress. For example, studies in *Arabidopsis thaliana* (L.) Heynh show that phenanthrene triggers widespread changes in the transcriptome, particularly affecting genes associated with photosynthesis, oxidative stress response, and hormone signaling [11]. In plant response to abiotic stress, genes encoding various antioxidant enzymes and antioxidants show significant up-regulation [12]. Furthermore, metabolites act as central mediators in plant adaptive responses to stress. Research indicates that key metabolites, including amino acids and secondary compounds like polyphenols and flavonoids, contribute to the enhancement of plant abiotic stress tolerance [13,14]. Li et al. (2024) used a combined transcriptomic-metabolomic approach to show that phosphorus enhances *Salix caprea* L. root adaptation to cadmium stress and its phytoremediation efficacy; this is achieved by boosting superoxide dismutase/catalase (CAT) activity, promoting flavonoid/ascorbic acid (AsA) biosynthesis, activating JA signaling, and up-regulating cadmium transporter genes [15]. A short-term hydroponic experiment demonstrated that *Salix viminalis* L. combats phenanthrene stress through the up-regulation of glutathione and amino acid biosynthesis, coupled with enhanced carbohydrate metabolism [16]. Collectively, insights from transcriptomic and metabolomic studies provide a more comprehensive understanding of the complex mechanisms underlying plant responses to abiotic stresses, such as exposure to PAHs. However, these studies have largely focused on short-term exposure to a single-PAH, such as phenanthrene or pyrene, and hydroponic systems, whereas the mechanisms governing root adaptation to long-term PAHs stress in soil remain poorly understood. In particular, the integrated physiological, transcriptomic, and non-targeted metabolomic responses of *Buchloe dactyloides* (Nutt) Engelm under long-term soil PAHs contamination have not yet been systematically characterized. *B. dactyloides* is a highly tolerant herbaceous plant and can be used for the remediation of PAHs contaminated soils [17]. It was hypothesized that long-term PAHs contamination in soil would trigger coordinated physiological, transcriptional, and metabolic adjustments in *B. dactyloides* roots, thereby enhancing stress adaptation and remediation potential. Therefore, this study aimed to comprehensively investigate the adaptive responses of *B. dactyloides* roots to long-term PAHs stress from physiological, transcriptomic, and non-targeted metabolomic perspectives, thereby providing new insights into its defense mechanisms and phytoremediation potential.

## 2. Results

### 2.1. Physiological Indicators

**Figure 1:**
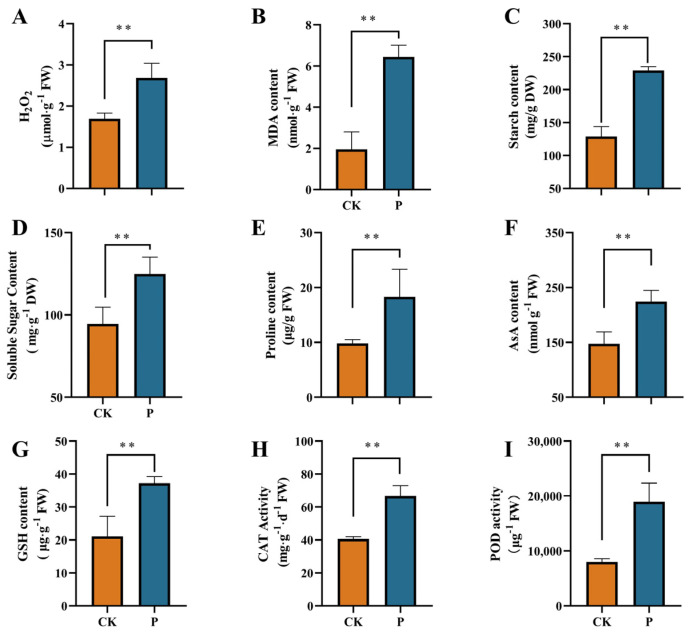
Effect of PAHs treatments on H_2_O_2_ content (**A**), MDA content (**B**), starch content (**C**), soluble sugar content (**D**), proline content (**E**), AsA content (**F**), GSH content (**G**), CAT activity (**H**), and POD activity (**I**). The bar indicates mean ± SE (*n* = 3). **, *p* < 0.05. FW: fresh weight. DW: dry weight. CK, normal soil; P, soil with PAHs.

Hydrogen peroxide (H_2_O_2_), Malondialdehyde (MDA), starch, soluble sugar, proline, AsA, glutathione (GSH), CAT, and peroxidase (POD) all showed an increasing trend under PAHs exposure (Figure 1).

### 2.2. Transcriptomic Analysis

#### 2.2.1. RNA-Seq

**Figure 2:**
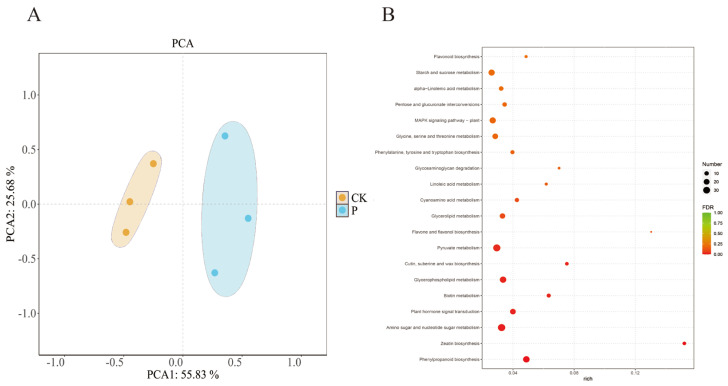
Transcriptomic analysis under PAHs treatments (**A**). KEGG enrichment analysis of differentially expressed genes (**B**). CK, normal soil; P, soil with PAHs.

A total of 42,877,897,184 raw data and 40,889,453,080 clean data were obtained through sequencing. The mean values of Q20 and Q30 were 98.06% and 94.59%, respectively (Table 1). The principal component analysis (PCA) results showed that PC1 and PC2 cumulatively explained 80.51% of the total sample variance (55.83% and 25.68% for PC1 and PC2, respectively) (Figure 2A).

#### 2.2.2. Differentially Expressed Genes (DEGs)

In this study, FPKM was utilized as a metric to measure transcript and gene expression levels. A total of 4083 DEGs (2395 upregulated and 1688 downregulated) were identified through screening based on a significance threshold of *p*-adjust < 0.05 and |log2FC| ≥ 1.

Through Kyoto Encyclopedia of Genes and Genomes (KEGG) enrichment analysis, 20 significantly enriched pathways in roots exposed to PAHs were identified (Figure 2B). Among the top 20 enriched pathways, flavonoid biosynthesis, starch, and sucrose metabolism, alpha-Linolenic acid metabolism, pentose, and glucuronate interconversions, MAPK signaling pathway, glycine, serine, and threonine metabolism, phenylalanine, tyrosine, and tryptophan biosynthesis, glycosaminoglycan degradation, linoleic acid metabolism, cyanoamino acid metabolism, glycerolipid metabolism, flavone and flavonol biosynthesis, pyruvate metabolism, cutin, suberine and wax biosynthesis, glycerophospholipid metabolism, biotin metabolism, plant hormone signal transduction, amino sugar and nucleotide sugar metabolism, and zeatin biosynthesis showed significant enrichment under PAHs stress. This preliminary observation suggests that flavonoid biosynthesis, carbohydrate metabolism, and amino acid synthesis may play potentially key roles in the detoxification response of *B*. *dactyloides* to PAHs stress.

#### 2.2.3. Quantitative Real-Time Polymerase Chain Reaction (qRT-PCR)

**Figure 3:**
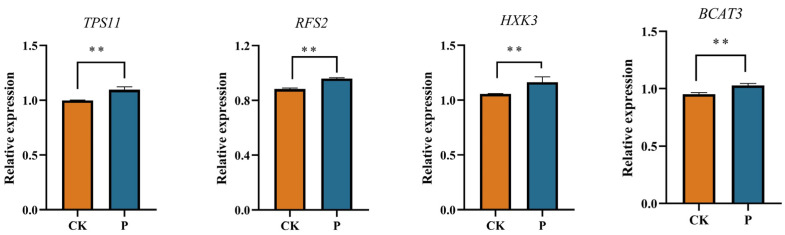
Validation of differential expression genes by qRT-PCR. Reference gene expression was set as 1, and the relative expression levels were calculated for several genes. CK, normal soil; P, soil with PAHs. Trehalose-6-phosphate synthase (*TPS11*); raffinose synthase (*RFS2*); Hexokinase (*HXK3*); Branched-Chain Amino Acid Aminotransferase (*BCAT3*). **, *p* < 0.05.

To verify the accuracy of the transcriptome data, four genes from different pathways were randomly selected for qRT-PCR. The results showed that all the candidate genes showed similar expression patterns with the transcriptome data (Figure 3). Therefore, the results of the analysis of transcriptome data were reliable.

### 2.3. Metabolomic Analysis

Non-targeted metabolomics analysis was performed using UHPLC-QE-MS, where metabolites detected in both positive and negative ion modes were combined for subsequent analyses. The orthogonal partial least squares-discriminant analysis (OPLS-DA) analysis revealed significant differences between treatment groups (Figure S1). These results indicate significant differences among the treatment groups. Metabolites with a *p* of <0.05 and variable importance in projection (VIP) ≥ 1 on the *t*-test were considered to be differentially accumulated metabolites (DAMs) between two groups.

**Figure 4:**
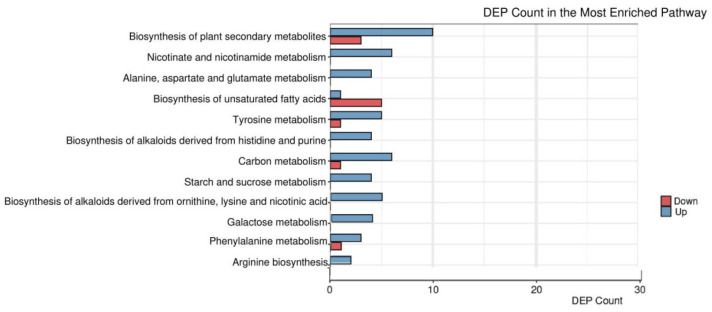
Metabolomic analyses under PAHs treatments.

The KEGG enrichment results for DAMs were displayed in Figure 4. In addition, a portion of DAMs showed enrichment in pathways such as alanine, aspartate and glutamate metabolism, Biosynthesis of unsaturated fatty acids, tyrosine metabolism, carbon metabolism, starch and sucrose metabolism, galactose metabolism, and arginine biosynthesis.

### 2.4. Integrated Analysis of Transcriptome and Metabolome Under PAHs Stress

**Figure 5:**
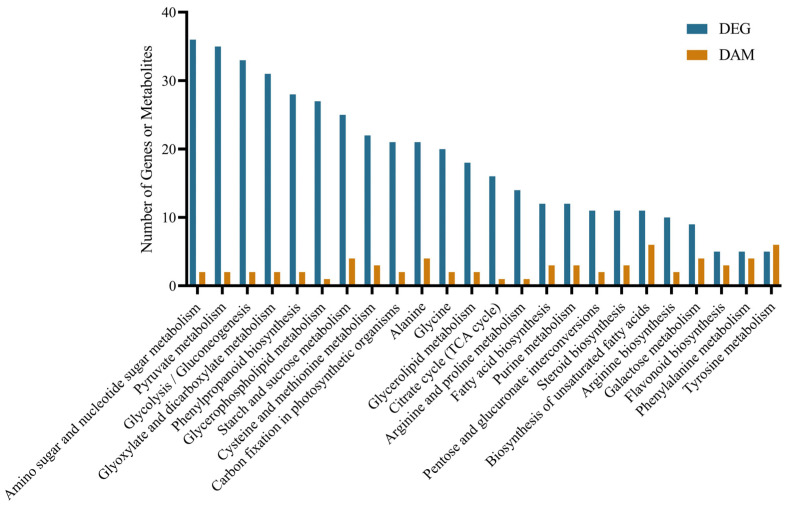
KEGG enrichment analysis of DEGs and DAMs of the same pathway in CK vs. P.

The KEGG enrichment analysis of DAMs and DEGs in CK and P identified 24 pathways in which both genes and metabolites responded differentially to PAHs stress (Figure 5). Integrated transcriptomic and metabolomic analyses further suggested that the root defense response to PAHs stress involves flavonoid biosynthesis, starch and sucrose metabolism, and amino acid synthesis.

#### 2.4.1. Biosynthesis of Flavonoid

**Figure 6:**
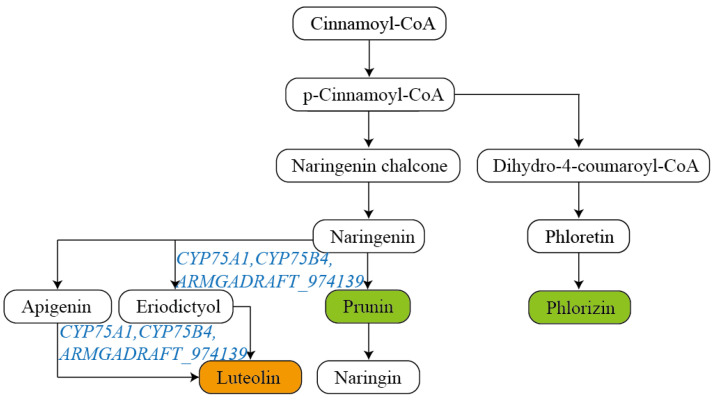
Analysis of partial flavonoid biosynthesis pathway in CK vs. P. The rectangles represented metabolites. Green and brown colors of rectangles represent up and down-regulation, respectively. Blue genes represent down-regulation.

A total of 5 DEGs and three DAMs were enriched in the flavonoid biosynthetic pathway (Figure 6). In the flavonoid biosynthetic pathway, all DEGs showed inhibition under PAHs stress, including *CYP75A1* and *CYP75B4*. The expression of luteolin was upregulated under PAHs stress. Notably, in the naringin and phlorizin biosynthetic pathway, where p-cinnamoyl-CoA acts as the precursor, the levels of prunin and phlorizin were increased, while no corresponding DEGs were enriched. These changes may contribute to the accumulation of secondary metabolites and help enhance the antioxidant capacity and stress adaptation of plants under PAHs exposure.

#### 2.4.2. Metabolism of Carbohydrates

**Figure 7:**
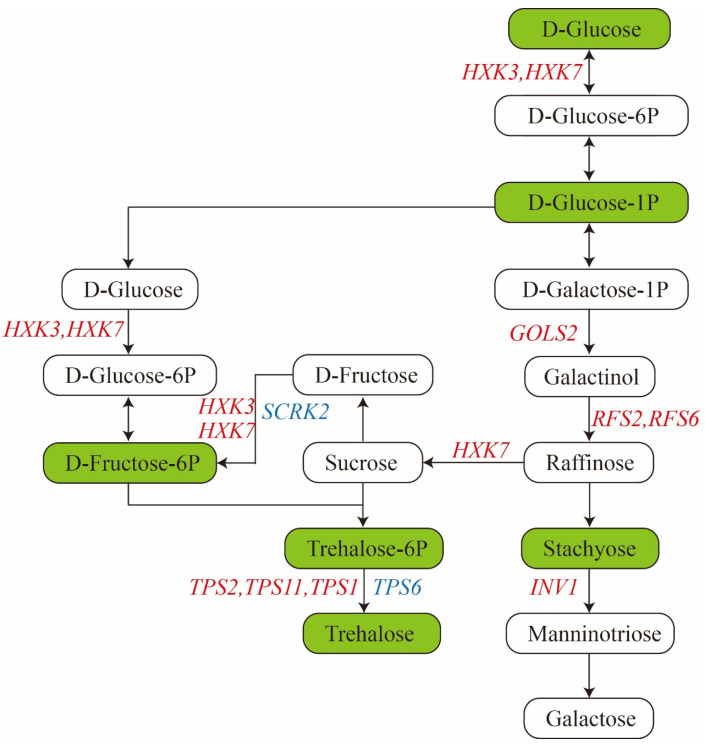
Analysis of partial carbohydrates metabolism pathway in CK vs. P. The rectangles represented metabolites. Green color of rectangles represents up-regulation. Red and blue genes represent up and down-regulation, respectively.

A total of 11 DEGs and 5 DAMs were enriched in the galactose metabolism (Figure 7). Compared with the control samples, the plants subjected to 70 days PAHs showed significant accumulation of trehalose and stachyose. In the present study, galactinol synthase (*GOLS2*), trehalose-6-phosphate synthase (*TPS1*, *TPS2*, *TPS11*), Hexokinase (*HXK3*, *HXK7*), β-fructofuranosidase (*INV1*), and raffinose synthase (*RFS2*) were relatively upregulated at 70 days PAHs exposure. Thus, the upregulation of several sugars and related genes would be in response to the osmotic regulation and high energy requirements under PAHs stress.

#### 2.4.3. Amino Acid Synthesis

**Figure 8:**
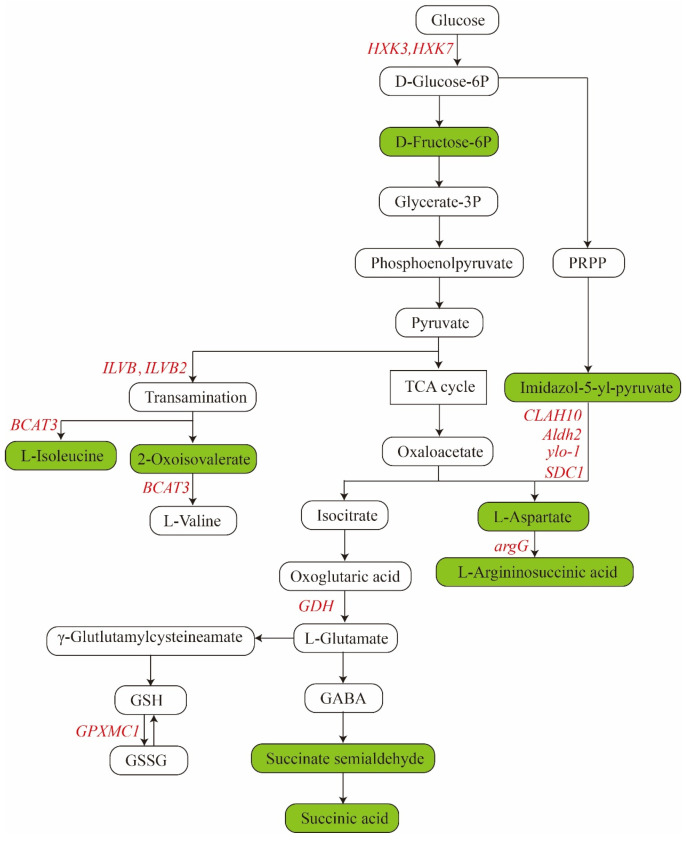
Analysis of partial Amino acid synthesis pathway in CK vs. P. The rectangles represented metabolites. Green color of rectangles represents up-regulation. Red and blue genes represent up and down-regulation, respectively.

A total of 11 DEGs and 8 DAMs were enriched in the amino acid synthesis (Figure 8). In the amino acid synthesis, all DEGs showed inhibition under PAHs stress, including glutathione peroxidase (*GPXMC1*), NADP-specific glutamate dehydrogenase (*GDH*), Serine decarboxylase (*SCD1*), Argininosuccinate Synthase (*argG*), Aldehyde Dehydrogenase (*Aldh2*), Acetolactate Synthase (*ILVB*, *ILVB2*) and Branched-Chain Amino Acid Aminotransferase (*BCAT3*). The levels of isoleucine, 2-Oxoisovalerate, succinic acid, succinate semialdehyde, L-Argininosuccinate, L-aspartate, D-Fructose-6P, and Imidazol-5-yl-pyruvate was upregulated under PAHs stress. Overall, PAHs exposure appears to reprogram amino acid metabolism in roots, which may facilitate plant adaptation to stress by modulating nitrogen allocation, energy metabolism, and cellular redox and osmotic homeostasis.

## 3. Discussion

In this study, a 70 days experiment was conducted to elucidate the changes in roots under PAHs stress. Kumar et al. (2023) reported that low-ring PAHs (e.g., phenanthrene and pyrene) exhibit acute toxicity, whereas high-ring PAHs (e.g., benzo[a]pyrene) possess stronger carcinogenic properties [18]. Given their ubiquitous distribution and severe pollution levels [19], this study focuses on phenanthrene, pyrene, and benzo[a]pyrene.

Damage to plants caused by abiotic stress is primarily mediated through an imbalance in ROS dynamics, resulting in a burst of ROS. Exposure to PAHs induces the production of ROS, including superoxide anion (O_2_^−^), H_2_O_2_, and hydroxyl radicals (·OH), which in turn damage subcellular structures and organelles. Among these, H_2_O_2_ represents the predominant ROS during oxidative stress in plant cells and tissues [3]. In this study, the roots produced a large amount of oxidative stress, manifested by higher levels of H_2_O_2_ and MDA. The accumulation of H_2_O_2_ is a key factor contributing to cell death upon exposure to PAHs [3]. To counteract the oxidative stress caused by ROS, plants activate a complex network of regulatory mechanisms including various biochemical reactions (e.g., enzymatic antioxidant) and molecular processes (e.g., gene expression regulation) to adapt to stressful environments [20]. In the context of these mechanisms, the antioxidant system (which includes enzymatic antioxidants and non-enzymatic antioxidants) is an important defense mechanism of plants to cope with abiotic stress [3]. The antioxidant enzyme system includes key players such as CAT and POD, which have a broad spectrum of actions. In our study, PAHs stress was associated with increased oxidative stress in roots, which is consistent with previous research results that accumulation of PAHs leads to excessive production of ROS, especially H_2_O_2_ (Figure 1). The decomposition of H_2_O_2_ into water and oxygen is primarily mediated by the CAT, which is found in cellular peroxisomes. Under PAHs stress, the enzyme activity of CAT in roots showed an increasing trend, and in the transcriptome data, antioxidant enzyme-related genes of CAT showed an upward trend (Figure S2). These results suggest that CAT may play an important role in the plant response to PAHs stress. Proline accumulation is considered a key response, through which a reduction in cellular water potential is achieved, leading to limited water loss, maintained osmotic pressure, and alleviated lipid peroxidation [21]. Additionally, the increase in soluble sugar and starch levels may reflect metabolic adjustments under PAHs stress.

Under abiotic stress, plants produce a range of secondary metabolites. Key among these are small molecules such as ASA, flavonoids, and GSH, which play an important role in scavenging reactive oxygen species and collectively form a crucial non-enzymatic antioxidant system, essential for maintaining growth and development under stress [15]. Flavonoid-related genes and metabolites have been shown to enhance reactive oxygen species scavenging capabilities [22]. For instance, exogenous flavonoid application has been reported to improve drought tolerance in peanut by modulating the homeostatic balance of reactive oxygen species within guard cells [23]. Liu et al. (2022) found that arbuscular mycorrhizal fungi alleviated oxidative damage in trifoliate orange under water stress by inducing flavonoid synthesis [24]. Flavonoids can act as antioxidants to scavenge reactive oxygen species generated during cold stress, thereby improving plant cold tolerance by regulating stress-responsive genes and pathways [15]. AcCHS5, a flavonoid synthesis gene, increases antioxidant enzyme activity and reduces reactive oxygen species accumulation by coordinating flavonoid biosynthesis [25]. In this study, the flavonoid biosynthetic pathway was significantly enriched upon exposure to PAHs, suggesting that it may be involved in the plant response to PAHs stress. In this study, the contents of prunin and phlorizin under PAHs stress were significantly higher than those in the control group (Figure 6). Prunin is a lipid-soluble compound characterized by antioxidant activity, which enables it to reduce oxidative damage [26,27]. This makes it an important defense molecule for plant responses to stress like salinity-waterlogging stress [26]. Phlorizin is considered a strong antioxidant that directly scavenges free radicals [28]. Therefore, prunin and phlorizin might contribute to alleviating oxidative damage in plants under PAHs stress by scavenging ROS. The observed decrease in luteolin may be attributed to reduced expression of *CYP75A1* and *CYP75B4*. Ascorbic acid, a prevalent plant small molecule, acts as a key antioxidant by directly reacting with diverse reactive oxygen species, thereby playing an indispensable role in the cellular defense system [29]. The findings suggested that increased AsA levels contributed to plant tolerance against the ROS burst (Figure 1). Furthermore, the upregulation of two key genes, *MDA3* and *MIOX4*, was observed under PAHs stress. These genes encode central regulators of the ascorbic acid–glutathione cycle and the myo-inositol pathway, respectively, and play a critical role in AsA synthesis and regeneration [30]. This further suggests that plants respond to PAHs stress through the coordinated action of multiple pathways to maintain AsA levels.

Under adverse environmental conditions, sugars play a key role in plant osmoregulation, ROS scavenging, and provision of large amounts of energy through metabolic processes to maintain cellular energy status [31]. Trehalose and its derivatives function as both metabolic resources and structural components in plant cells, while also acting as an osmoprotectant for plants experiencing stress conditions [32]. Kaya et al. reported that trehalose offers protection against oxidative stress in plants [33]. All differential metabolites involved in starch and sucrose metabolism and galactose metabolism were up-regulated under PAHs exposure (Figure 7). Additionally, the observed increases in soluble sugars and starch were align with the transcriptomic and metabolomic signatures of altered carbohydrate metabolism. The upregulation of *TPS2*, *TPS11* and *TPS1*, coupled with accumulation of trehalose, starch and soluble sugars, shows consistent physiological, transcriptomic and metabolomic signatures, suggesting that carbohydrate metabolism is an important metabolic adjustment of *B. dactyloides* under PAHs exposure. PAHs stress induced an alteration in carbon metabolism, as evidenced by the accumulation of starch and soluble sugars. The increased levels of trehalose and stachyose, along with the upregulation of *GOLS2*, *RFS2*, *TPS*, *HXK3*, *HXK7*, and *INV1*, indicate an enhanced sugar metabolic and signaling response that may contribute to osmotic adjustment and stress adaptation. These coordinated physiological, transcriptomic, and metabolomic changes imply that PAHs exposure triggers carbon reallocation toward soluble and protective sugars, potentially contributing to osmoprotection and stress tolerance.

Amino acids serve as precursors for the synthesis of numerous defensive metabolites in organisms under environmental stress, including various secondary metabolites such as glutathione and alkaloids [6]. In addition to their structural roles, certain amino acids contribute to plant stress resilience by participating in osmoregulation and protein synthesis. In this research, the levels of succinic semialdehyde and succinic acid increased significantly under exposure to PAHs. The secretion of succinic acid by roots has been identified as a potential plant strategy to mitigate PAHs stress [34]. Addition of succinic acid reduced ROS activity and alleviated *Scirpus triqueter* L. plasma membrane damage [35]. Aspartate, as the central node of plant nitrogen metabolism and amino acid metabolism, is pivotal in stress responses by regulating antioxidant systems and signal transduction pathways [36]. Research indicates that exogenous aspartic acid mitigates salt stress in wheat, primarily through the reduction of ROS, thereby alleviating growth inhibition [37].

Lipids are essential biomolecules that serve critical roles in cellular metabolism and energy storage. Increased levels of lipid metabolites also contribute to greater accumulation of contaminants [38]. Previous research has found that naphthalene, pyrene, and benzo(a)pyrene disrupt phospholipid metabolism in *Nitzschia brevirostris*, causing damage to lipids and cell membranes [39]. In this study, 14 pathways related to lipid metabolism (including glycerophospholipid metabolism, glycerolipid metabolism, linoleic acid metabolism, α-linolenic acid metabolism, fatty acid biosynthesis, arachidonic acid metabolism, and unsaturated fatty acid biosynthesis) were identified, indicating that PAHs significantly affected the lipid metabolism of plant roots. In addition, GPXMC1 and 5-Oxoicosatetraenoic acid, both derived from arachidonic acid metabolism, were elevated under PAHs stress and may play an important role in plant defense responses (Figure S3) [40,41]. These polyunsaturated fatty acids contribute to the maintenance of root cell membrane fluidity, thereby enhancing osmotic stress tolerance and reducing plasma membrane damage [42].

Jasmonate (JA), derivatives of lipids, act as vital signaling compounds in diverse plant stress responses and development. Previous studies have shown that JA mediates responses to various stresses including osmotic, drought, and heavy metal stresses [43]. It is noteworthy that exogenous JA mitigates plant damage under abiotic stress by activating multiple protective mechanisms, including the up-regulation of antioxidant enzymes and secondary metabolites, and the scavenging of ROS [44,45]. Under PAHs stress, three JAZ genes showed higher expression levels (Figure S4). This suggests that plants may enhance their positive role in the stress response to PAHs by up-regulating genes involved in JA transduction.

## 4. Materials and Methods

### 4.1. Materials

Soil samples (0–20 cm depth) were collected from an agricultural field in Beijing, China (40°0′27″ N, 116°15′22″ E) in 2022 (total rainfall 253.7 mm, average temperature 18 °C). The soil was homogenized, air-dried, and sieved through a 2 mm mesh. Its key physicochemical properties are summarized in Table 2. Based on previous studies, the PAHs stressed soils containing phenanthrene, pyrene, and benzo[a]pyrene (Macklin, Shanghai, China), were prepared as follows [46]. First, phenanthrene (0.20 g), pyrene (0.20 g), and benzo[a]pyrene (0.10 g) were dissolved in 500 mL of acetone and thoroughly mixed into 0.20 kg of soil. The mixture was then placed in a fume hood to allow complete acetone evaporation. After evaporation, it was blended with an additional 0.20 kg of clean soil. Finally, this spiked soil was progressively mixed into the remaining soil to yield a total of 20 kg of uniformly PAHs contaminated soil. Prior to the experiment, the concentrations of phenanthrene, pyrene and benzo[a]pyrene were determined, resulting in 3.40 ± 0.02 mg kg^−1^, 3.61 ± 0.05 mg kg^−1^, and 2.64 ± 0.06 mg kg^−1^ in the PAHs stressed soils, respectively.

### 4.2. Plant Treatment

The pot experiment was conducted on *B. dactyloides* in a greenhouse under natural daylight and temperatures of 25–35 °C. *B. dactyloides* seedlings were transplanted into plastic pots (three plants per pot) containing 0.7 kg of soil, with or without added PAHs, for a total of 20 pots. Soil moisture was maintained at 60% field capacity by regular weighing and replenishment with sterile distilled water. Pot positions were randomized weekly. After a 70 days growth period, roots were carefully harvested to avoid damage, immediately frozen in liquid nitrogen, and stored for subsequent analysis. For metabolomic analysis, roots from five randomly selected plants within the same treatment were pooled as one biological replicate, yielding six biological replicates per treatment. For transcriptomic analysis and subsequent physiological measurements, the six metabolomic pools were further combined pairwise to generate three pooled samples per treatment, which were used as biological replicates.

### 4.3. RNA Extraction, Library Construction, and Sequencing

After 70 days of treatment, *B. dactyloides* roots were taken for transcriptome sequencing. Each treatment was performed in three biological replicates. Total RNA was extracted using Trizol reagent kit (Invitrogen, Carlsbad, CA, USA) according to the manufacturer’s protocol. The Agilent2100 Bioanalyzer (Agilent Technologies, Palo Alto, CA, USA) was used to perform quality inspection on the library, and then detect the total concentration of the library and the effective concentration of the library. Total RNA was checked using RNase free agarose gel electrophoresis. After total RNA was extracted, eukaryotic mRNA was enriched by Oligo(dT) beads. Then the enriched mRNA was fragmented into short fragments (300 bp). Using RNA as a template, use 6-base random primers and reverse transcriptase to synthesize the first strand of cDNA, and use the first strand cDNA as a template to synthesize the second strand cDNA. After extraction, purification, and library preparation, libraries were sequenced using paired-end (PE) next-generation sequencing on an Illumina HiSeq platform. No reference genome or annotation version was used. The assembled transcriptome was used as the reference for read alignment and transcript quantification using RSEM 1.3.3.

### 4.4. Annotation and Analysis of Transcriptome Data

Raw sequencing reads were quality-filtered to obtain clean reads, which were then aligned and quantified using FPKM. Differential gene expression analysis was performed using DESeq2 for group comparisons. Transcripts with a false discovery rate (FDR) < 0.05 and an absolute fold change ≥ 2 were defined as DEGs. These DEGs were subsequently subjected to functional enrichment analysis based on Gene Ontology (GO) and the KEGG databases.

### 4.5. qRT-PCR

Total RNA was extracted from *B. dactyloides* tissues using the RNA Easy Fast Plant Tissue Kit (TransGen Biotech, Beijing, China) according to the manufacturer’s instructions. RNA concentration and purity were determined using a NanoDrop One spectrophotometer (Thermo Scientific, Waltham, MA, USA), and integrity was verified by agarose gel electrophoresis with ethidium bromide staining. cDNA was synthesized from 1 µg of total RNA using the Easy Script^®^ One-Step gDNA Removal and cDNA Synthesis Kit (TransGen Biotech). qRT-PCR was performed on a Bio-Rad CFX96 system using gene-specific primers (Table 3) and the TB Green^®^ Premix Ex Taq™ Kit (Takara Biomedical Technology, Beijing, China). Each treatment was performed in three biological replicates and evaluated amplification efficiencies.

### 4.6. Liquid Chromatography and Mass Spectrometry

Six biological replicates were analyzed for metabolomics. Bison grass root samples that have been quick-frozen in liquid nitrogen are ground and weighed into 200 mg, mixed with 70% methanol (containing 2-chloro-L-phenylalanine), and vortexed for 45 s. After grinding, ultrasonic for 15 min. Centrifuge at 12,000 rpm for 10 min, and filter the supernatant with a 0.22 μm membrane. Root metabolites were detected by ultra-high performance liquid chromatography (Thermo Fisher Scientific, Waltham, MA, USA, UPLC) tandem mass spectrometry (Thermo Fisher Scientific, Waltham, MA, USA, MS/MS). A quality control (QC) sample was prepared by pooling equal aliquots of supernatant from each experimental sample. Raw mass spectrometry data were converted to mzXML format using ProteoWizard and subsequently processed with an in-house R pipeline based on the XCMS package for peak detection, extraction, alignment, and integration. Metabolite annotation was performed by matching the acquired MS/MS spectra against an in-house spectral database (BiotreeDB).

Positive and negative ion mode data were analyzed separately. To visualize group separation, OPLS-DA was performed using the R package v4.3.1 ‘mixOmics’ (Figure S1). Differential metabolites were screened by combining univariate Student’s *t*-tests (*p* < 0.05) and VIP scores (VIP ≥ 1) from the OPLS-DA model. The goodness of fit and predictive ability of the model were evaluated using *R*^2^*Y* and Q^2^ values. The robustness of the model was validated by permutation testing to exclude overfitting. The explanatory and predictive abilities of the model were evaluated using *R*^2^*X*, *R*^2^*Y*, and Q^2^ values. In positive ion mode, the OPLS-DA model yielded *R*^2^*X* = 0.276, *R*^2^*Y* = 0.999, and Q^2^ = 0.736. In negative ion mode, the OPLS-DA model yielded *R*^2^*X* = 0.27, *R*^2^*Y* = 0.996, and Q^2^ = 0.613. Metabolites meeting both criteria were defined as DAMs and subsequently subjected to KEGG pathway enrichment analysis.

### 4.7. Determination of Starch, Soluble Sugar, Malondialdehyde, Hydrogen Peroxide, Proline, Antioxidant Metabolites, and Antioxidant Enzymes

The activities of CAT and POD, and the levels of AsA, H_2_O_2_, starch, soluble sugar, MDA, GSH, and proline detected using kit (BC0205, BC0090, BC1230, BC3595, BC0700, BC0035, BC0025, BC1175, and BC0290, Solarbio, Beijing, China) following the manufacturer’s protocol, respectively. Each treatment was performed in three biological replicates.

### 4.8. Statistical Analysis

The normality of the datasets was assessed using the Shapiro–Wilk test, and homogeneity of variance was evaluated using Levene’s test before statistical analysis (SPSS Statistics 24, Armonk, NY, USA). The variables were analyzed using the independent samples *t*-test. PCA was performed on the experimental parameters using R version 4.3.1 software (DESeq2). Graphs were generated using GraphPad Prism 8 (GraphPad Software, San Diego, CA, USA).

## 5. Conclusions

This study employed an integrative analysis of physiological measurements, transcriptomics, and metabolomics to elucidate the defense mechanisms of *B. dactyloides* under PAHs exposure. The results indicate that plants exhibit active defense responses to PAHs stress. Specifically, *B. dactyloides* alleviates PAHs-induced oxidative and lipid peroxidation stress by enhancing CAT and POD activities and by accumulating proline, AsA, and GSH; it also modulates amino acid synthesis and flavonoid biosynthesis. In addition, plants improve the antioxidant capacity of roots by upregulating *INV1*, *RFS2*, *TPS1,* and *HXK3* in galactose metabolism. Trehalose, starch, soluble sugar, and stachyose were the predominant carbohydrates that accumulated in roots under PAHs stress, suggesting they may contribute to energy supply and stress tolerance during the response to PAHs exposure. Finally, the roots promoted the synthesis of polyunsaturated fatty acids by upregulating the expression of genes related to α-linolenic acid metabolism, fatty acid biosynthesis, arachidonic acid metabolism, as well as the increased of *GPXMC1* and the content of 5-Oxoicosatetraenoic acid. Collectively, these physiological, transcriptomic, and metabolomic signatures indicate that adjustments to carbohydrate metabolism, enhanced antioxidant defenses (including CAT, POD, GSH, and AsA), changes in amino acid metabolism, increased flavonoid biosynthesis, and remodeling of lipid metabolism are key components of the adaptive response of roots to long-term PAHs exposure. We propose to investigate the roles of hormones and their crosstalk in modulating the physiological, transcriptomic, and metabolic responses of roots to PAHs exposure.

## Figures and Tables

**Table 1:** Overview of transcriptome sequencing of complementary DNA from *Buchloe dactyloides*.

Sample	Raw Data (bp)	Clean Data (bp)	Q20 (%)	Q30 (%)	N (%)
CK1	7,781,429,244	7,415,276,290	98.11	94.62	0.000418
CK2	8,144,939,698	7,766,180,962	97.99	94.41	0.000422
CK3	6,494,891,728	6,192,671,872	97.98	94.35	0.00043
P1	6,537,424,502	6,237,826,912	98.05	94.57	0.000411
P2	7,539,837,096	7,191,094,140	98.07	94.62	0.000416
P3	6,379,374,916	6,086,402,904	98.18	94.95	0.000409

**Table 2:** Properties of soil in this study.

	pH	SOM	TN	TP	TK	AP	AK	AN	CEC
Content	8.42 ± 0.08	18.86 ± 0.51	0.93 ± 0.01	0.94 ± 0.06	18.03 ± 0.63	35.99 ± 0.59	173.78 ± 17.43	101.52 ± 2.79	11.53 ± 0.34

SOM: soil organic matter, g·kg^−1^; TN: total nitrogen, g·kg^−1^; TP: total phosphorus, g·kg^−1^; TK: total potassium, g·kg^−1^; AP: available phosphorus, mg·kg^−1^; AK: available potassium, mg·kg^−1^; AN: Alkali-hydrolyzable nitrogen, mg·kg^−1^; CEC: cation exchange capacity, cmol·kg^−1^.

**Table 3:** The gene specific primers used for qRT-PCR.

Name	Forward Primer (5′-3′)	Reverse Primer (5′-3′)
*HXK3*	AGAGGTGAAAGCGAAAGAAA	GGGAGAGGAGGAGAAATAGAA
*TPS11*	CCTGGGTGCTTCTGATTTCT	GAGCCACATTCTGTCGGTAA
*BCAT3*	GAATACAGCAGGCAAGGCA	CCTCCGTATCGTCGTCAAG
*RFS2*	GTCGGTGAAGAGGCAGTCA	AAGCCAGGGAACCACAACT
*Actin*	CCCAATCTATGAAGGCTACGC	CGGCAGTGGTTGTGAAAGAGTA

## Data Availability

Dataset available on request from the authors. The raw data supporting the conclusions of this article will be made available by the authors on request.

## Supplementary Materials

The following supporting information can be downloaded at: https://www.mdpi.com/article/10.3390/plants15152315/s1, Figure S1. OPLS-DA score plots and permutation tests for the *B. dactyloides* root metabolome in positive (left) and negative (right) ion modes across different treatments; Figure S2. Analysis of Peroxisome in CK vs. P. Red and green genes represent up and down-regulation, respectively; Figure S3. Analysis of lipid metabolism pathway in CK vs. P; Figure S4. Analysis of partial plant hormone signal transduction pathways in CK vs. P.

## Abbreviations

The following abbreviations are used in this manuscript:

*Aldh2*	Aldehyde Dehydrogenase
*argG*	Argininosuccinate Synthase
*AsA*	Ascorbic acid
BCAT3	Branched-Chain Amino Acid Aminotransferase
CAT	Catalase
DAMs	Differentially accumulated metabolites
DEGs	Differentially expressed genes
GDH	NADP-specific glutamate dehydrogenase
GO	Gene Ontology
*GOLS2*	Galactinol synthase
*GPXMC1*	Glutathione peroxidase
GSH	Glutathione
H_2_O_2_	Hydrogen peroxide
*HXK3*, *HXK7*	Hexokinase
*INV1*	β-fructofuranosidase
JA	Jasmonate
KEGG	Kyoto Encyclopedia of Genes and Genomes
*ILVB*, *ILVB2*	Acetolactate Synthase
OPLS-DA	Orthogonal partial least squares-discriminant analysis
PAHs	Polycyclic aromatic hydrocarbons
POD	Peroxidase
qRT-PCR	Quantitative real-time polymerase chain reaction
*RFS2*	Raffinose synthase
ROS	Reactive oxygen species
*SCD1*	Serine decarboxylase
*TPS1*, *TPS2*, *TPS11*	Trehalose-6-phosphate synthase
