# Supplementary material for: Integrated Physiological, Transcriptomic, and Metabolomic Analyses Reveal the Adaptive Response of Buchloe dactyloides to Polycyclic Aromatic Hydrocarbon Stress
Source: Plants (Basel). 2026 Jul 28;15(15):2315. doi: 10.3390/plants15152315 (PMC13468443; doi:10.3390/plants15152315)
Supplement: Supplementary file 1 [file plants-15-02315-s001.zip › plants-4420357-supplementary.pdf]

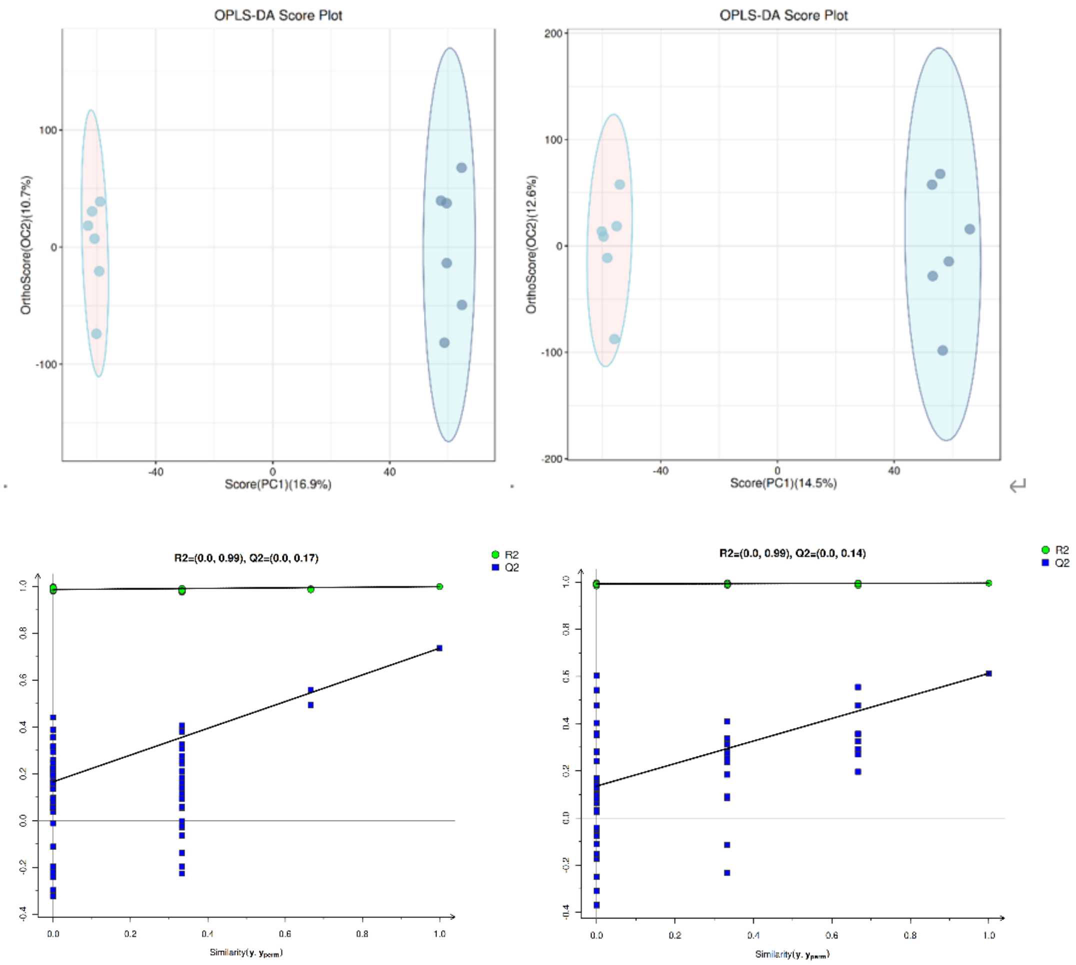

Figure S1. OPLS-DA score plots and permutation tests for the *B. dactyloides* root metabolome in positive (left) and negative (right) ion modes across different treatments.

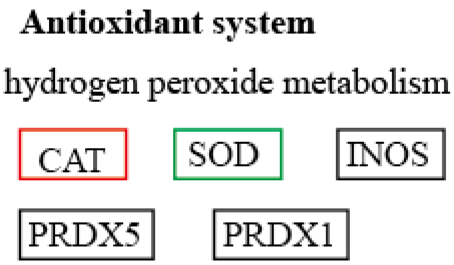

Figure S2. Analysis of Peroxisome in CK vs P. Red and green genes represent up and down-regulation, respectively.

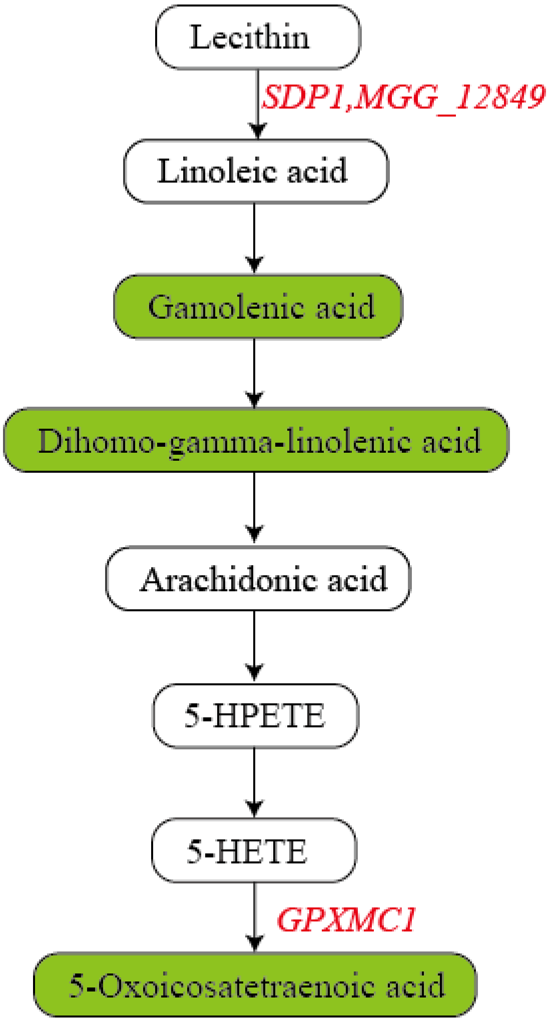

Figure S3. Analysis of lipid metabolism pathway in CK vs P. The rectangles represented metabolites. Green and brown colors of rectangles represent up and down-regulation, respectively. Red and blue genes represent up and down-regulation, respectively.

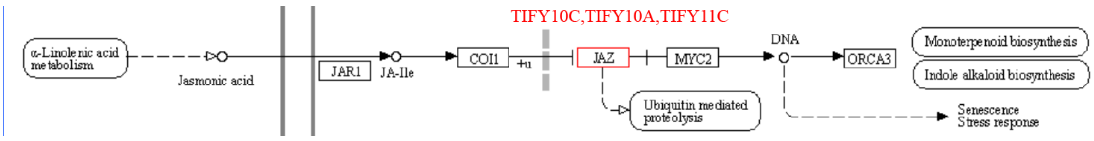

Figure S4. Analysis of partial plant hormone signal transduction pathways in CK vs P. Red and blue genes represent up and down-regulation, respectively.
